# Pressure-Dependent Thermal and Mechanical Behaviour of a Molecular Crystal of Bromine

**DOI:** 10.3390/molecules29194744

**Published:** 2024-10-08

**Authors:** Madhavi H. Dalsaniya, Deepak Upadhyay, Paras Patel, Prafulla K. Jha, Krzysztof Jan Kurzydłowski, Dominik Kurzydłowski

**Affiliations:** 1 Faculty of Materials Science and Engineering, Warsaw University of Technology, 02-507 Warsaw, Poland; madhavi.dalsaniya.dokt@pw.edu.pl (M.H.D.); krzysztof.kurzydlowski@pw.edu.pl (K.J.K.); 2 Faculty of Mathematics and Natural Sciences, Cardinal Stefan Wyszyński University in Warsaw, 01-038 Warsaw, Poland; deepakupadhyay888@gmail.com; 3 Department of Physics, Faculty of Science, The Maharaja Sayajirao University of Baroda, Vadodara 390002, Gujarat, India; pparas727@gmail.com (P.P.); prafullaj@yahoo.com (P.K.J.)

**Keywords:** bromine, molecular crystals, quasi-harmonic approximation, thermal properties, dissociation, mechanical properties

## Abstract

This study investigates the pressure-dependent thermal and mechanical properties of solid bromine through density functional theory (DFT) calculations used in conjunction with the quasi-harmonic approximation (QHA). At ambient pressure, bromine crystallizes as a molecular crystal of *Cmca* symmetry. Previous studies have indicated that upon compression, this polymorph should undergo a bandgap closure at 80 GPa followed by a phase transition to a nonmolecular phase at 90 GPa. By employing QHA, we model the lattice vibrations and calculate the free energy, thermal expansion, and specific heat capacities of solid molecular bromine over a temperature range from 0 to 1000 K and pressures up to 90 GPa. Furthermore, mechanical properties such as bulk modulus and elastic constants are also analyzed. The results reveal the significant impact that pressure has on the thermal properties, mechanical stability, and dynamical stability of a molecular crystal. These findings contribute to a deeper understanding of such systems under extreme conditions, potentially guiding future experimental and theoretical investigations.

## 1. Introduction

The accurate modelling of the thermodynamical characteristics of a material is important for both fundamental science and practical applications, as it provides insight into how materials behave under varying pressure and temperature conditions [1,2]. Both of these parameters influence the stability of compounds, as well as the equilibrium of chemical reactions. While pressure of an order 10^9^ Pa (=1 GPa) can induce many interesting phenomena, such as phase transition, superconductivity, and exotic quantum states, it can also lead to structural changes in materials, including the formation of new crystalline structures, changes in electronic properties, and even the emergence of new forms of matter like superionic phases [3,4]. High temperatures lead to increased atomic vibrations contributing to anharmonicity [5], which significantly influences atomic fluctuations, thermal expansion, and compressibility in solids [6]. Importantly, when both variables are controlled, exotic transition can be observed. For example, the formation of superionic ice occurs when water molecules dissociate, allowing hydrogen ions to move freely through an oxygen lattice [7,8].

Quasi-harmonic approximation (QHA) is a highly effective theoretical approach to studying the thermo-mechanical properties of materials [9,10,11,12]. This method provides valuable insights into thermal lattice fluctuations at high temperatures and the zero-point energy contribution at low temperatures [13]. One of the key benefits of QHA is its ability to predict thermodynamic properties at a constant pressure, which is crucial in understanding material behaviour under realistic conditions. This method has been successfully applied to study the thermodynamic properties of molecular solids such as solid water [14] and oxygen [15], among others [16,17]. These studies have provided accurate predictions of thermal expansion and phase stability. Understanding the mechanical properties of molecular crystals is a critical aspect of their application as smart materials [18].

**Figure 1:**
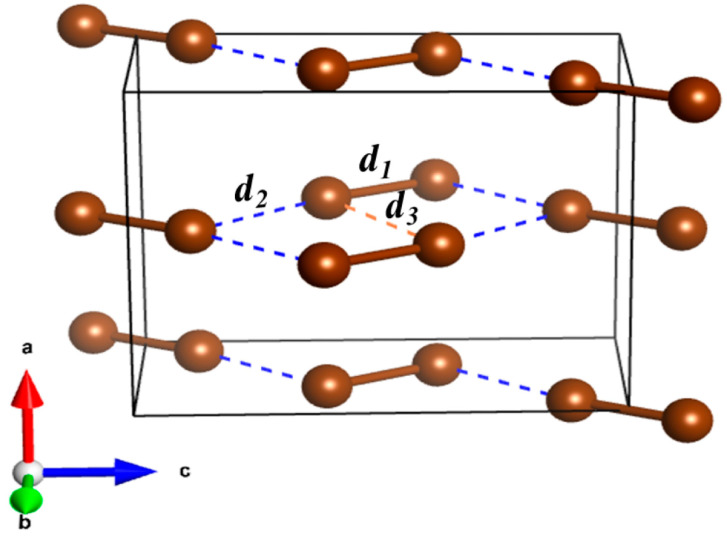
Crystal structure of the Br_2_ *Cmca* phase at 0 GPa. The intramolecular single bond is denoted as *d*_1_, while *d*_2_ (blue dashed lines) and *d*_3_ (orange dashed line) represent the intermolecular interactions between the molecules. The cell vectors (***a***, ***b*** and ***c***) are depicted by red/green/blue arrows.

However, there is still a significant gap regarding the thermodynamic properties of many other molecular solids, especially those that are electron-rich, such as halogen molecules. Their unique behaviour under high-pressure conditions has not been thoroughly studied, limiting our understanding of their thermodynamic characteristics. Although, in ambient conditions (1 atm, 293 K), bromine is liquid, it solidifies at 266 K into a molecular crystal exhibiting *Cmca* symmetry and four molecules in the unit cell (*Z* = 4). The same structure is obtained upon room-temperature compression of the liquid below 1 GPa [19]. Within this crystal, diatomic molecules (Br_2_) are bound by a single bond (*d*_1_ = 2.30 Å in Figure 1) with weak intermolecular (van der Waals) interactions (*d*_2_ and *d*_3_) linking the molecules within the ***bc*** plane. 

Due to the electronic similarity between bromine and elemental hydrogen, the pressure-induced phase transition sequence of the former has been the subject of several experimental and theoretical studies [20,21,22]. Recent experimental studies indicate that the molecular *Cmca* phase of bromine is stable at pressures up to at least 81 GPa [20]. Above this pressure, experiments have revealed the appearance of incommensurate structures existing between 81 and 112 GPa. These polymorphs are most probably intermediate phases occurring along the transition from the molecular *Cmca* structure to the nonmolecular *Immm* phase, which is predicted to be the ground state structure of bromine between 90 and 128 GPa [21]. Theory predicts further phase transitions occurring between nonmolecular phases: *Immm* to *I4/mmm* at 128 GPa, and finally from *I4/mmm* to a monoatomic *fcc* crystal at 188 GPa. An analogous phase transition sequence is observed in compressed iodine [23,24,25]. DFT calculations based on the generalized-gradient approximation (GGA) predict the closure of the electronic band gap of bromine to occur in the molecular *Cmca* phase at 42.5 GPa [22]. However, more advanced hybrid DFT calculations suggest that the band-gap closure for this phase occurs at a significantly higher pressure of 80 GPa [21].

In this study, we focus on the high-pressure properties of solid bromine in its molecular phase (*Cmca* symmetry) within its predicted thermodynamic stability range (1 atm ≈ 0 GPa to 90 GPa). Our research employs hybrid DFT calculations (utilizing the HSE06 functional [26]) combined with QHA to calculate the thermal and mechanical properties of this system. This methodology involves conducting phonon calculations at several (8–10) cell volumes around the equilibrium volume in the harmonic approximation, followed by applying an equation of state (see Supplementary Materials for a more detailed description). This process allows us to determine thermal expansion coefficients and other thermodynamic properties (like the Gibbs free energy, Helmholtz free energy, Grüneisen parameter, and bulk modulus) of solid molecular bromine. 

## 2. Results

### 2.1. Vibrational Properties under Pressure

**Figure 2:**
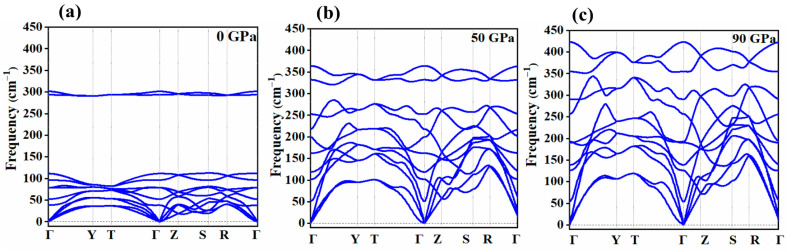
Phonon dispersion curve (blue lines) of the *Cmca* phase of bromine at (**a**) 0 GPa (**b**) 50 GPa, and (**c**) 90 GPa.

First, we discuss the vibrational properties of the *Cmca* phase of bromine and give a detailed examination of its thermodynamical and mechanical characteristics. Understanding the dynamic stability of a system under varying pressure conditions is crucial, as it provides insights into how the system behaves in response to changes in external forces. To investigate the dynamical stability of the *Cmca* structure, we computed the phonon spectrum along the high-symmetry directions in reciprocal space (Γ—Y—T—Γ—Z—S—R; for the notation of the high-symmetry points of the Brillouin zone, see [27]). The results are depicted in Figure 2. Phonons play a significant role in influencing various material properties. Therefore, to accurately determine these properties, it is essential to understand the complete phonon band structure. 

As can be seen in Figure 2, compression leads to the stiffening of the phonon structure, i.e., an increase in phonon frequencies and the slope in reciprocal space of the acoustic modes. Such behaviour, which stems from a pressure-induced increase in intermolecular interactions, is often found in molecular crystals subject to compression [28,29,30]. Importantly, we did not find any imaginary (unstable) vibrational modes in the studied pressure range. This testifies to the dynamical stability of the *Cmca* phase at ambient conditions and pressures up to 90 GPa, corroborating recent experimental findings [20]. At the centre of the Brillouin zone (Γ-point), the vibrational modes can be decomposed into irreducible representation with the following symmetries: 2B_1u_ + 2B_2u_ + B_3u_ + A_u_ + B_1g_ + B_2g_ + 2A_g_ + 2B_3g_. The total number of 12 vibrational modes corresponds directly to the 3N degrees of freedom, where N represents the number of atoms in the primitive cell. In the case of bromine, there are four atoms in the primitive cell, resulting in 3N = 12 vibrational modes. Among these modes, B_1u_, B_2u_, and B_3u_ are three acoustic modes that are associated with the translational motion of the crystal and have zero frequency at the Γ-point. Additionally, six Γ-point vibrational modes are Raman active: B_1g_ + B_2g_ + 2B_3g_ + 2A_g_. Five of them (1B_1g_, 2B_3g_, 2A_g_) were monitored in high-pressure experiments [19,31,32].

**Figure 3:**
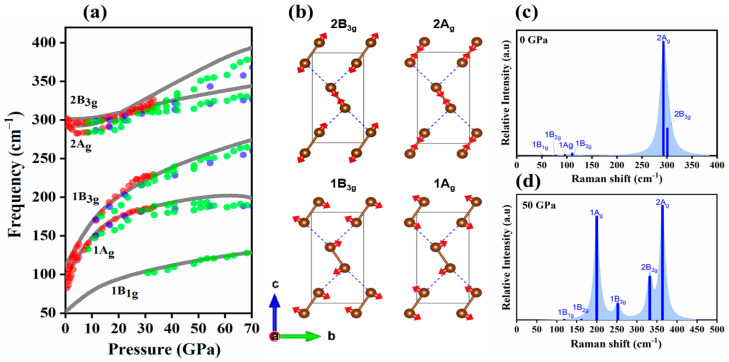
(**a**) Raman frequencies for the *Cmca* phase obtained in this work (HSE06, grey lines) and experiment (dots, red—ref. [19], green—ref. [28], blue—ref. [29], together with (**b**) the visualization of the atomic movements (red vectors) in the B_3g_ and Ag modes and the calculated Raman spectra (blue lines) of bromine at (**c**) 0 GPa and (**d**) 50 GPa.

As can be seen in Figure 3a, the calculated frequencies of the Raman-active vibrations agree very well with the experimental results. In particular, DFT modelling reproduces the frequency crossover between the 2A_g_ and 2B_3g_ stretching modes observed above 20 GPa, as well as the drastic hardening of the librational 1A_g_ and 1B_3g_ modes, which, upon compression to 30 GPa, exhibit an increase in frequency of over 100 cm^−1^. These two modes can be described as hindered rotations taking place within the ***bc*** plane (Figure 3b), which hosts the closest intermolecular contacts between Br_2_ molecules (see Figure 1). It is for this reason that these modes exhibit higher frequencies and more pronounced pressure dependence compared to the B_1g_ mode, which consists of rotations taking place in the plane perpendicular to the ***bc*** plane.

The Raman spectrum modelled for the *Cmca* phase shows that the 1B_1g_ and 1B_2g_ modes have negligible intensity at 0 GPa (Figure 3c), offering an explanation for why they are not observed experimentally at ambient pressure. Upon compression, their intensity grows, as shown by the modelled Raman spectrum at 50 GPa (Figure 3d). This, in turn, is in accordance with fact that the 1B_1g_ mode is observed experimentally above 30 GPa. In accordance with the experiment in [32], we found a pressure-induced increase in the intensity of the 1A_g_ mode. As can be seen in Figure 3d, the large intensity of this mode leads to the obscuration of the band originating from the 2B_2g_ vibration, explaining why it is not observed in high-pressure experiments.

### 2.2. Thermodynamical Properties under Pressure

**Figure 4:**
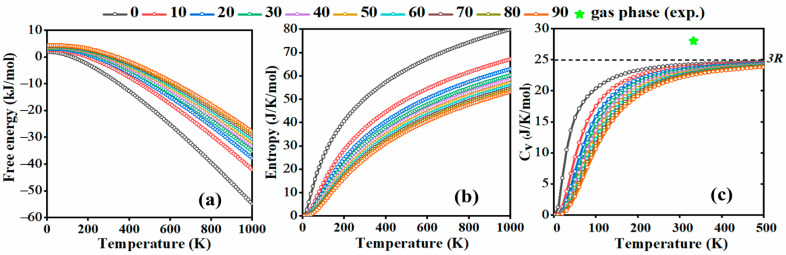
Calculated thermal properties of solid bromine (*Cmca* structure) as a function of temperature from 0 to 90 GPa: (**a**) free energy, (**b**) entropy, (**c**) heat capacity at constant volume (C_V_) (star—heat capacity for gaseous bromine, green—ref. [37]).

Free energy, heat capacity, and entropy are fundamental thermodynamic properties essential in understanding the behaviour of solids as functions of temperature and pressure. Free energy represents the amount of work that a system can perform, with Helmholtz free energy (F), defined as F = U − TS (where U is the internal energy and S is entropy) being relevant at constant volume. Gibbs free energy (G), defined as G = H − TS (where H is enthalpy), is applicable at constant pressure. Heat capacity measures the amount of heat required to raise the temperature of a given system by one degree Celsius. There are two main types: heat capacity at constant volume C_V_, expressed as $C_{V}={\left(\frac{\partial U}{\partial T}\right)}_{V}$, and heat capacity at constant pressure C_P_, given by $C_{V}={\left(\frac{\partial H}{\partial T}\right)}_{P}\;$. Entropy quantifies the number of possible microscopic configurations corresponding to a macroscopic state. In our study, the thermal properties of solid molecular bromine, including free energy, heat capacity, and entropy, are calculated from phonon frequencies at various pressures, as shown in Figure 4. We note that for the calculated properties, only the vibrational term has been included; this is, however, the main contribution to this function up to at least 70 GPa, as bromine remains a semiconductor up to this pressure.

The results in Figure 4c indicate that C_V_ increases with temperature but decreases with increasing pressure. At low temperatures, it exhibits the T^3^ behaviour predicted by the Debye law, which describes how heat capacities of solids vary at low temperatures due to phonon vibrations. However, as the temperature increases, this T^3^ dependence fails, and the heat capacities begin to approach the Dulong–Petit limit (3R ≈ 24.94 J/(mol∙K)). Upon compression of the *Cmca* phase of bromine, higher temperatures are required to reach the Dulong–Petit limit, as indicated by the decrease in the heat capacity at 500 K from 24.08 J/(mol∙K) to 21.85 J/(mol∙K) as the pressure increases from 0 to 90 GPa. This might be a signal of the increase in anharmonic contributions [33], which would be in accordance with the postulated pressure-induced increase in the strength of intermolecular interactions in the *Cmca* phase of bromine [21]. The QHA method partially incorporates anharmonic effects by allowing volume-dependent phonon frequencies, which accounts for the thermal expansion of the crystal [34,35]. However, there are more accurate methods for treating anharmonicity, which provide a better description of anharmonic phenomena beyond the quasi-harmonic regime [36]. We note that the modelled decrease in heat capacity upon compression is mostly a vibrational effect, as only this contribution is taken into account in our calculations. However, it is possible that the underlying mechanism is connected with changes in the electronic structure (e.g., a decrease in the bandgap). While this is an interesting point for future research, it lies beyond the scope of the current work.

**Figure 5:**
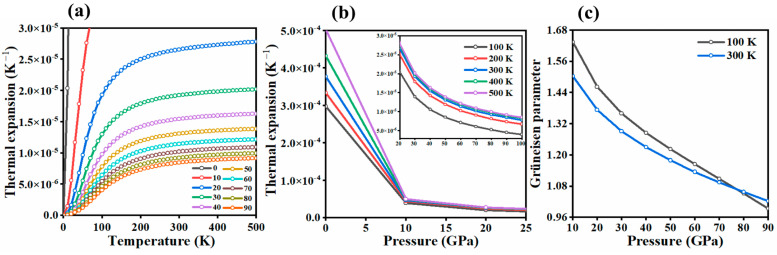
Variation in the thermal expansion coefficient for Br_2_ in the *Cmca* structure as a function of (**a**) temperature (**b**) pressure together with the (**c**) Grüneisen parameter.

In addition, we have calculated the thermal expansion coefficient (α) for solid bromine in the *Cmca* molecular phase (Figure 5). This coefficient reflects the variation in lattice volume with respect to changes in pressure or temperature and is defined as how much the volume of a material changes as a function of the temperature at constant pressure, typically expressed as $\alpha =\frac{1}{V}{\left(\frac{\partial V}{\partial T}\right)}_{P}$, where V is the volume and T is the temperature, and the derivative is taken at constant pressure. The value of α calculated for the *Cmca* phase at the ambient pressure (1 atm) and below bromine’s melting temperature (200 K) is 333 × 10^−6^ K^−1^, comparable to what is found for molecular crystals of organic substances (with a mean value of 168.8 × 10^−6^ K^−1^ [38]), but considerably smaller than the thermal expansion coefficient of weakly bound noble gas crystals (1000 × 10^−6^ K^−1^ as found for solid krypton around 75 K [39]).

Upon compression, the value of α drastically decreases and becomes much less temperature-dependent (Figure 5b). The large pressure-driven decrease in the thermal expansion coefficient is line with findings that pressure has a more pronounced effect on the volume of solids compared to temperature [40]. At 10 GPa and 200 K, α equals 45 × 10^−6^ K^−1^—a value close to that found for alloys in ambient conditions (e.g., 25 × 10^−6^ K^−1^ for aluminium [41]). The Grüneisen parameter [42], which is an important thermodynamic quantity that elucidates the impact of pressure on a given material and indicates the strength of anharmonic effects within the vibrating lattice, is shown in Figure 5c for Br_2_ Cmca at different pressure.

### 2.3. Mechanical Properties under Pressure

The elastic constants of materials indicate how a material reacts to applied stress/strain or how much stress/strain is needed to achieve a specific deformation. The change in these constants under pressure can provide valuable insights into the mechanics behind phase transitions and the mechanical stability of the crystal structure [43,44]. These properties provide key parameters for evaluating the brittleness, stiffness, hardness, and eventually structural stability of the materials. Specifically, the orthorhombic system has nine independent elastic constants (*C_ij_*). In order for the system to be mechanically stable, these constants must obey the Born stability criteria [45,46], defined by the following relationships:

$$C_{ortho\;}=\left[\begin{array}{cccccc} C_{11} & C_{12} & C_{13} & . & . & .\\ . & C_{22} & C_{23} & . & . & .\\ . & . & C_{33} & . & . & .\\ . & . & . & C_{44} & . & .\\ . & . & . & . & C_{55} & .\\ . & . & . & . & . & C_{66} \end{array}\right]$$

*C*_ii_ > 0, *C*_ii_ + *C*_jj_ − 2*C*_ij_ > 0,

*C*_11_ + *C*_22_ + *C*_33_ + 2(*C*_12_ + *C*_13_ + *C*_23_) > 0.

**Figure 6:**
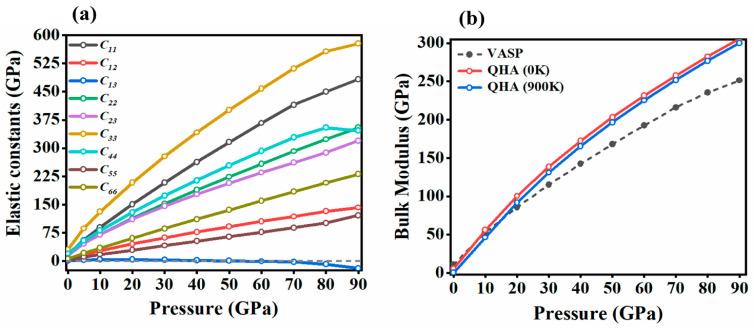
The pressure dependence of (**a**) the calculated elastic constants (at 0 K) and (**b**) the bulk modulus, with a comparison between VASP and QHA for bromine.

The stability criteria under various pressures indicate that the Br_2_ *Cmca* phase remains mechanically stable up to 90 GPa. As shown in Figure 6a, the elastic stiffness coefficients increase linearly with pressure. However, the *C*_13_ coefficient starts to decrease and becomes negative at 60 GPa (Table 1). We also observe the softening of the C_44_ constants above 80 GPa. Given that the bandgap closure of bromine is predicted to occur at this pressure [21], the decrease in *C*_44_ could be connected with this change in the electronic structure (i.e., the softening of the crystal due to the onset of metallicity). This result is consistent with the calculations of Duan et al., who, through GGA calculation, found similar softening at elastic constants upon the bandgap closure in the *Cmca* phase of bromine [47]. However, their less accurate computational method led to a much lower pressure for the metal-to-insulator transition (55 GPa) and, consequently softening, of the elastic components at a smaller compression—see Figure S1 for a comparison of their and our results.

Additionally, The Voigt–Reuss–Hill (VRH) approximation [48,49] is employed to determine the pressure-dependent isotropic bulk modulus (*B*), shear modulus (*G*), and Young’s modulus (*Y*) by the following formulas:

*B_V_* = [*2*(*C*_12_ + *C*_13_ + *C*_23_) + *C*_11_ + *C*_22_ + *C*_33_]/9

*B_R_* = 1/[(*S*_11_ + *S*_22_ + *S*_33_) + 2(*S*_12_ + *S*_13_ + *S*_23_)]

*G_V_* = (*C*_11_ + *C*_22_ + *C*_33_ − *C*_12_ − *C*_13_ − *C*_23_)/15 + (*C*_44_ + *C*_55_ + *C*_66_)/5

*G_R_* = 15/4[(*S*_11_ + *S*_22_ + *S*_33_) − 4(*S*_12_ + *S*_13_ + *S*_23_) + 3(*S*_44_ + *S*_55_ + *S*_66_)]

*B_H_* = (*B_V_* + *B_R_*)/2

*G_H_* = (*G_V_* + *G_R_*)/2

**Figure 7:**
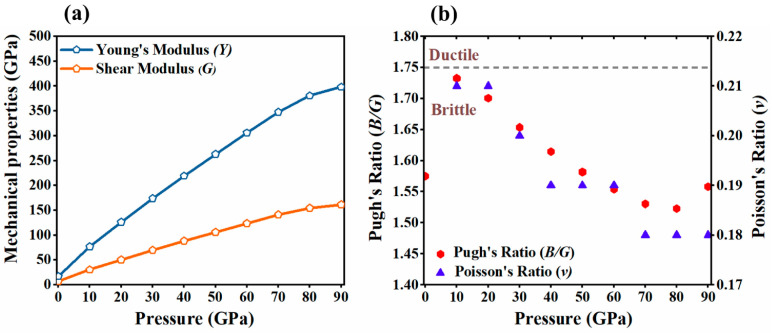
The pressure dependence of (**a**) Young’s modulus (*Y*) and the shear modulus (*G*) and (**b**) the variations in Pugh’s ratio (*B*/*G*) and Poisson’s ratio (*v*) for bromine at 0 K.

*Y_H_* = (9 × *G* × *B*)/(3*B_H_* + *G_H_*)

where *B_R_* and *G_R_* are the Reuss bulk/shear modulus, *B_V_* and *G_V_* are the Voigt bulk/shear modulus, *B_H_* and *G_H_* are the Hill bulk/shear modulus, and *S_ij_* (*i*, *j* = 1, 2, and 3) are the elastic compliance constants, obtained from the inverse of the matrix of the calculated elastic constants. The results calculated using the above equations are presented in Table 2 and depicted in Figure 6b and Figure 7a. At 0 GPa, the bulk modulus *B*_0_, estimated from elastic constants, is approximately 11.8 GPa, which is close to, but higher than, the 5.5 GPa obtained via QHA calculations (as shown in Figure 6b). Both values are close to those found experimentally for isostructural molecular crystals of chlorine (*B*_0_ = 8.3 GPa) and iodine (*B*_0_ = 7.0 GPa) [28,50]. In this context, the zero-pressure bulk modulus recently reported for the *Cmca* phase of bromine (*B*_0_ = 22.0 GPa) seems to be erroneously high, probably a result of fitting the equation of state to a limited number of pressure points [20]. The calculated Shear and Young’s moduli indicate that at a pressure of 30 GPa, the bromine elastic modulus reaches a value typical of steel.

Figure 7a shows that the moduli increase with pressure, with Young’s modulus consistently being the highest, and the shear modulus, the lowest, across the pressure range of 0–90 GPa, suggesting that bromine becomes harder under high pressure. As shown in Figure 6b and Figure 7a, *B* increases rapidly with pressure and remains consistently larger than *G*, indicating that the shear modulus is the limiting stability parameter for the compound. 

According to Pugh [51], the shear modulus (G) represents resistance to plastic deformation, while the bulk modulus (*B*) indicates resistance to fracture. The ratio *B*/*G* is linked to a transition from ductility to brittleness. If *B*/*G* > 1.75, then the material is ductile; otherwise, it is brittle. The brittleness of bromine in its orthorhombic phase is suggested by a Pugh’s ratio (*B*/*G*) less than 1.75. The brittleness of the compound is further supported by a Poisson’s ratio below 0.26, as shown in Figure 7b and Table 2. Additionally, the negative Cauchy pressure, determined by the difference between the elastic constants *C*_12_ and *C*_44_, confirms this brittleness. A negative Cauchy pressure suggests that the material is brittle, whereas a positive value would indicate ductility. Thus, the negative value found in this case confirms that bromine is indeed brittle in its orthorhombic phase.

## 3. Methods

We utilized solid-state density functional theory (DFT) calculations in the framework of the hybrid HSE06 functional [26] with the inclusion of van der Waals corrections (D3-Grimme) [52,53], as implemented in VASP 6.3.0. [54,55]. This computer-intensive method was successfully used to model the ambient pressure crystal structure of bromine, as well as compression-induced distortions [21]. The projector-augmented wave method included the *4s*_2_ and *4p*_5_ states of Br atoms as valence electrons. We started by optimizing the molecular crystal of bromine across pressures ranging from 0 to 90 GPa in 10 GPa steps. The calculations were performed using a plane-wave basis set with a cutoff energy of 800 eV, and the convergence criterion for electronic minimization was 10^−8^ eV. Brillouin zone sampling was performed using the Monkhorst–Pack mesh with a k-point spacing of 2π × 0.033 Å^−1^ [56]. All structures were optimized until the forces on the atoms were below 1 meV/Å. For phonon dispersion calculations and elastic properties, we used a supercell approach with the finite displacement method, employing Phonopy software (version 2.18.0) [57] to calculate the force constants from a 3 × 5 × 3 supercell, consisting of 360 atoms. Phonon band structures were generated by interpolating phonon frequencies along q-point paths through high-symmetry points in the Brillouin zone of the primitive cell. The Raman scattering intensities were calculated using the finite displacement method, as implemented in Phonopy-spectroscopy code [58]. To determine the thermodynamic properties of bromine under pressure, we utilized the QHA approach [9] through an interface programme compatible with Phonopy. This approach allowed us to compute thermal properties such as free energy, heat capacity, and entropy, based on phonon frequencies using a 15 × 15 × 15 mesh grid in reciprocal space. Calculations were performed using 10 volume–energy data points, fitted to the Vinet equation of state (EoS) [59].

## 4. Conclusions

In summary, we calculated the thermal and mechanical properties of orthorhombic bromine with *Cmca* symmetry from 0 to 90 GPa using density functional theory, utilizing a hybrid functional with corrections for van der Waals interactions. The phonon dispersion curve indicates that the Br_2_ *Cmca* phase remains dynamically stable up to 90 GPa. We also found that bromine is mechanical stable up to this pressure. However, we observed the softening of the *C*_13_ and *C*_44_ elastic constants, possibly indicating the system transition towards a metallic state. Bromine in solid state is brittle under pressure up to 80 GPa, as evidenced by Pugh’s ratio *B*/*G* < 1.75 and Poisson’s ration ϑ < 0.26. The calculated bulk modulus of bromine is in accordance with those determined experimentally for isostructural crystals of iodine and chlorine. Additionally, using the quasi-harmonic approximation, we determined that the heat capacity of bromine at 0 GPa and 500 K was 24.08 J/(mol∙K), which approaches the Dulong–Petit limit. The thermal expansion coefficient (α) at the ambient condition (1 atm and 200 K) is 333 × 10^−6^ K^−1^. The principles and techniques used in this study can be extended to other molecular solids, providing a comprehensive framework for predicting and controlling material properties in various scientific and industrial applications.

## Figures and Tables

**Table 1:** Calculated elastic constants *C_ij_* (GPa) of solid bromine at 0 K.

P (GPa)	*C*_11_	*C*_12_	*C*_13_	*C*_22_	*C*_23_	*C*_33_	*C*_44_	*C*_55_	*C*_66_
0	16.3	4.4	1.0	16.7	15.6	31.0	19.8	3.4	5.9
5	55.4	16.8	2.9	46.9	46.6	86.6	53.8	10.6	20.9
10	89.5	27.3	3.9	71.0	70.4	131.3	81.0	17.3	34.8
20	151.0	45.5	3.9	113.7	111.1	208.6	129.2	29.1	60.9
30	208.4	61.9	3.1	152.1	145.8	278.2	173.3	41.2	86.2
40	263.2	77.2	1.9	188.8	177.4	341.9	214.6	53.0	111.2
50	315.6	91.5	0.6	223.9	207.0	401.6	254.2	64.8	135.8
60	366.6	105.2	−0.7	258.2	235.0	457.8	292.2	76.6	160.1
70	415.3	118.4	−1.9	291.8	261.8	511.5	328.6	88.5	184.2
80	449.7	132.3	−8.4	323.5	288.1	557.0	354.4	100.7	207.8
90	483.4	142.3	−19.3	354.4	319.9	577.8	346.5	121.3	230.6

**Table 2:** The elastic moduli of solid Br_2_ (*Cmca*), such as the bulk modulus (*B*), the shear modulus (*G*), Young’s modulus (*Y*), and Pugh’s ratio (*B*/*G*), are calculated at 0 K.

P (GPa)	*B* (GPa)	*G* (GPa)	*Y* (GPa)	*B*/*G*
0	10.9	6.9	17.2	1.6
10	53.1	30.6	77.1	1.7
20	85.7	50.4	126.4	1.7
30	115.0	69.5	173.7	1.7
40	142.2	88.1	219.1	1.6
50	167.8	106.1	263	1.6
60	192.4	123.8	305.9	1.6
70	216.0	141.1	347.7	1.5
80	235.5	154.6	380.7	1.5
90	251.2	161.2	398.4	1.6

## Data Availability

The data supporting the findings of this study are available on the Repository for Open Data server (https://repod.icm.edu.pl/ accessed on 13 September 2024) under the DOI number 10.18150/ZCN9DH.

## Supplementary Materials

The following supporting information can be downloaded at: https://www.mdpi.com/article/10.3390/molecules29194744/s1. Explanation of phonon calculations and QHA in the methodology section. Figure S1: Comparison of pressure-dependent elastic constants using PBE + D3 and HSE06 + D3 functionals, Figure S2: Density vs. pressure at 0 K and 300 K for bromine (0 to 90 GPa), Figure S3: Elastic constants and bulk modulus vs. density for bromine (0 to 90 GPa, 0 K), Figure S4: Young’s modulus (*Y*), shear modulus (*G*), Pugh’s ratio (*B*/*G*) and Poisson’s ratio (*ν*) vs. density for bromine (0 to 90 GPa, 0 K).
