# Supplementary material for: Pressure-Dependent Thermal and Mechanical Behaviour of a Molecular Crystal of Bromine
Source: Molecules. 2024 Oct 8;29(19):4744. doi: 10.3390/molecules29194744 (PMC11477802; doi:10.3390/molecules29194744)
Supplement: Supplementary file 1 [file molecules-29-04744-s001.zip › molecules-3226857-supplementary.pdf]

Supplementary Information

Pressure-Dependent Thermal and Mechanical Behaviour of a Molecular Crystal of Bromine

*Madhavi H. Dalsaniya[1,2], Deepak Upadhyay [2], Paras Patel[3], Prafulla K. Jha[3], Krzysztof Jan Kurzydłowski[1] and Dominik Kurzydłowski[2]**

[1] Faculty of Materials Science and Engineering, Warsaw University of Technology, Wołoska 141, 02-507, Warsaw, Poland.

madhavi.dalsaniya.dokt@pw.edu.pl, krzysztof.kurzydlowski@pw.edu.pl

2 Faculty of Mathematics and Natural Sciences, Cardinal Stefan Wyszyński University in Warsaw, 01-038 Warsaw, Poland.

deepakupadhyay888@gmail.com, d.kurzydlowski@uksw.edu.pl

3 Department of Physics, Faculty of Science, The Maharaja Sayajirao University of Baroda, Vadodara, Gujarat, India-390002.

pparas727@gmail.com, prafullaj@yahoo.com

Correspondence: d.kurzydlowski@uksw.edu.pl

Methodology

Phonon Calculations Using Phonopy

Phonon calculations were carried out using the supercell approach, with phonon frequencies derived from the force constants via the PHONOPY code. The thermal properties of bromine at constant volume were subsequently computed based on the phonon density of states as a function of frequency [1,2]. For more detailed information on the thermodynamical expression, refer to Section 2.2. (For more instructions on phonon calculations, visit this link: https://phonopy.github.io/phonopy/vasp.html).

Quasi-Harmonic Approximation (QHA)

The QHA was applied using the Phonopy framework to study thermodynamic properties such as Free energy, Thermal expansion, Bulk modulus and the Grüneisen parameter for bromine. QHA accounts for anharmonic effects by incorporating the volume dependence of phonon frequencies. For this calculation, at least five volume points are required for fitting. Phonon calculations for bromine were carried out using a 3×5×3 supercell over a pressure range of 0 to 90 GPa, in 10 GPa increments. A volume-energy data file was prepared accordingly and the resulting free energies at each volume were fitted to an equation of state to extract

thermodynamic properties as functions of both temperature and pressure [1]. More information on this process can be found here: https://phonopy.github.io/phonopy/qha.html#)

Mechanical properties

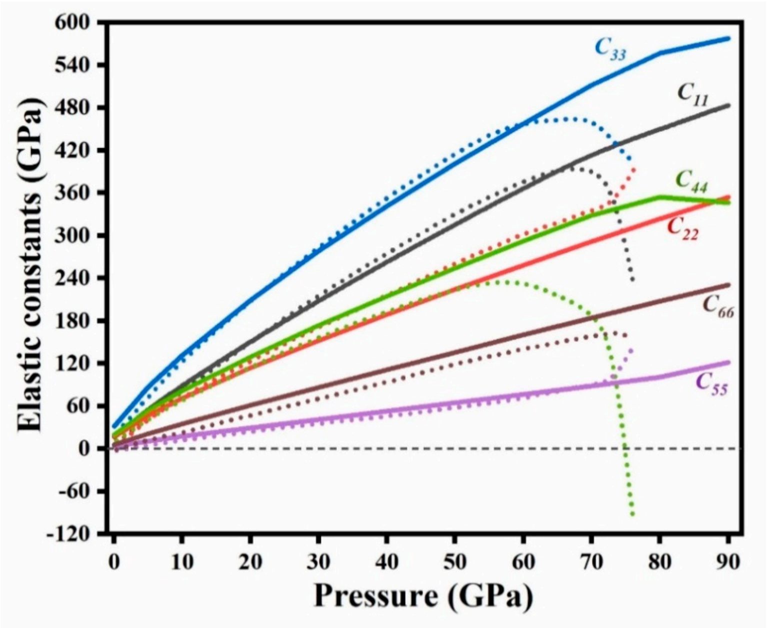

Figure S1. Comparison of pressure-dependent elastic constants using the PBE+D3 and HSE06+D3 functionals. The solid lines depict the current HSE06+D3 results, while the dotted lines indicate previously reported PBE+D3 data. [3].

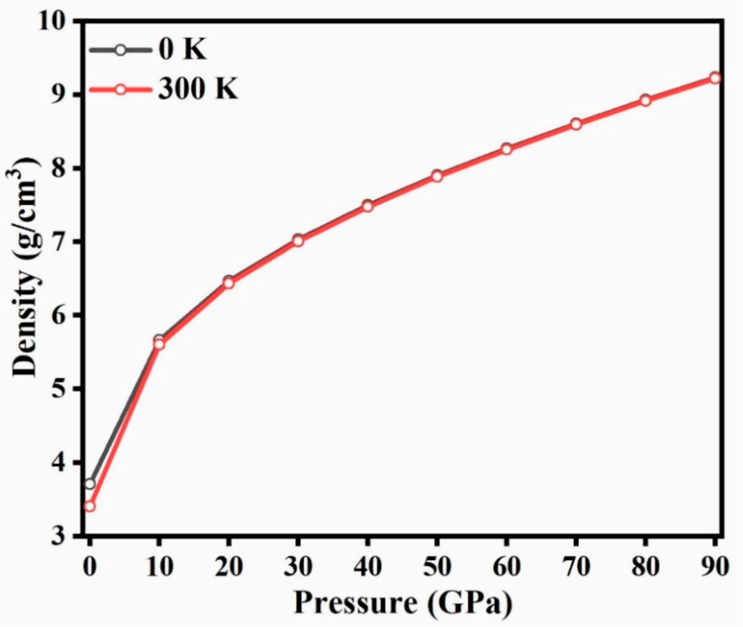

Figure S2. Calculated density variation of bromine as a function of pressure from 0 to 90 GPa.

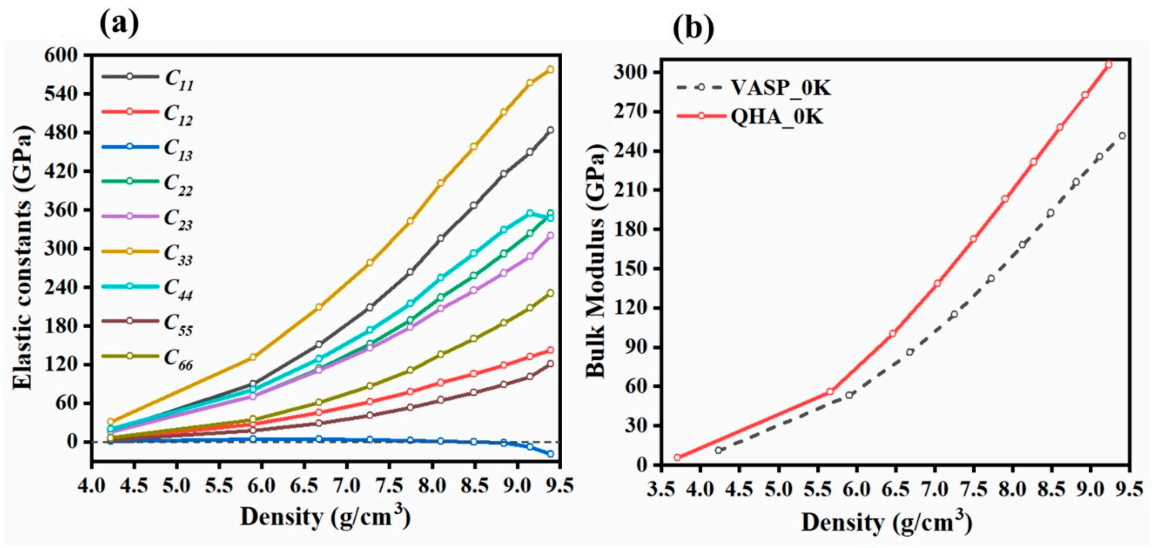

Figure S3. (a) Calculated elastic constants as a function of density at 0 K and (b) bulk modulus as a function of density for bromine, with a comparison between VASP and QHA calculations.

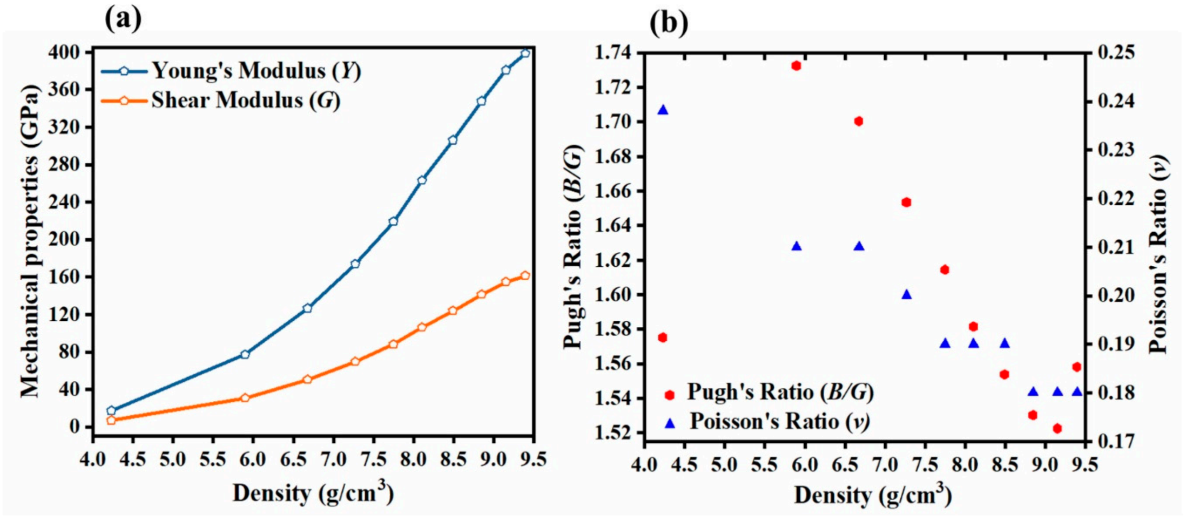

Figure S4. (a) Young's modulus (Y) and shear modulus (G) as a function of density and (b) the variation of Pugh's ratio (B/G) and Poisson's ratio (v) as a function of density for bromine at 0 K.

References :

1. Togo, A.; Chaput, L.; Tanaka, I.; Hug, G. First-Principles Phonon Calculations of Thermal Expansion in Ti_3SiC_2, Ti_3AlC_2, and Ti_3GeC_2. *Phys. Rev. B* **2010**, *81*, 174301, doi:10.1103/PhysRevB.81.174301.

2. Togo, A.; Tanaka, I. First Principles Phonon Calculations in Materials Science. *Scr. Mater.* **2015**, *108*, 1–5, doi:10.1016/j.scriptamat.2015.07.021.

3. Duan, D.; Liu, Y.; Ma, Y.; Liu, Z.; Cui, T.; Liu, B.; Zou, G. Ab Initio Studies of Solid Bromine under High Pressure. *Phys. Rev. B - Condens. Matter Mater. Phys.* **2007**, *76*, 1–8, doi:10.1103/PhysRevB.76.104113.
